# Immune Neuroendocrine Phenotypes in *Coturnix coturnix*: Do Avian Species Show LEWIS/FISCHER-Like Profiles?

**DOI:** 10.1371/journal.pone.0120712

**Published:** 2015-03-20

**Authors:** F. Nicolas Nazar, Bibiana E. Barrios, Pete Kaiser, Raul H. Marin, Silvia G. Correa

**Affiliations:** 1 Biological and Technological Investigations Institute (IIByT), National Scientific and Technical Research Council (CONICET) and National University of Cordoba, Cordoba, Argentina; 2 The Roslin Institute, University of Edinburgh, Easter Bush, Midlothian, Scotland, United Kingdom; 3 Clinical Biochemistry and Immunology Research Center (CIBICI), National Scientific and Technical Research Council (CONICET) and National University of Cordoba, Cordoba, Argentina; Institute of Zoology, CHINA

## Abstract

Immunoneuroendocrinology studies have identified conserved communicational paths in birds and mammals, e.g. the Hypothalamus-Pituitary-Adrenal axis with anti-inflammatory activity mediated by glucocorticoids. Immune neuroendocrine phenotypes (INPs) have been proposed for mammals implying the categorization of a population in subgroups underlying divergent immune-neuroendocrine interactions. These phenotypes were studied in the context of the LEWIS/FISCHER paradigm (rats expressing high or low pro-inflammatory profiles, respectively). Although avian species have some common immunological mechanisms with mammals, they have also evolved some distinct strategies and, until now, it has not been studied whether birds may also share with mammals similar INPs. Based on corticosterone levels we determined the existence of two divergent groups in *Coturnix coturnix* that also differed in other immune-neuroendocrine responses. Quail with lowest corticosterone showed higher lymphoproliferative and antibody responses, interferon-γ and interleukin-1β mRNA expression levels and lower frequencies of leukocyte subpopulations distribution and interleukin-13 levels, than their higher corticosterone counterparts. Results suggest the existence of INPs in birds, comparable to mammalian LEWIS/FISCHER profiles, where basal corticosterone also underlies responses of comparable variables associated to the phenotypes. Concluding, INP may not be a mammalian distinct feature, leading to discuss whether these profiles represent a parallel phenomenon evolved in birds and mammals, or a common feature inherited from a reptilian ancestor millions of years ago.

## Introduction

Immunoneuroendocrinology (INE) arises from the study of the interaction of cells, mediators and organs that belong to the immune, nervous and endocrine systems, respectively. The well characterized main axes that support the mentioned interactions in superior vertebrates such as birds and mammals are: the Hypothalamus-Pituitary-Adrenal (HPA) axis, the Sympathetic-Adrenergic axis and the Vagal-Cholinergic axis [1–9]. The HPA axis acts as a master regulator that controls various body processes, including immunity, mainly having an anti-inflammatory activity mediated by glucocorticoids (GC), such as corticosterone (CORT), and a pro-inflammatory activity dependent on dehidroepiandrosterone [3,10–13]. At a molecular level, classical hormones such as GC, prolactin and growth hormone can be produced by immune cells, whereas a variety of cytokines, originally described as immune cell products, are now known to be synthesized and released by a variety of glands and neuroendocrine tissues [14–17]. Numerous cytokines, such as interleukin-1β (IL-1β), act as endogenous regulators influencing HPA secretory axis activity. Cytokine receptors have been cloned, characterized, and localized to many neuroendocrine (among other) tissues [15,18].

Immune neuroendocrine phenotypes (INPs) have been proposed for mammals [19–21]. This notion implies the categorization of an undisturbed animal population in subgroups expressing different INE interaction patterns. Animals that correspond to one or other subpopulation consistently differ in (i) neuroendocrine mediator concentrations, (ii) hormonal receptor activity and expression density and (iii) cytokine levels that belong to pro- or anti-inflammatory profiles. These INPs have been relevant in the study of pathogenic mechanisms in LEWIS (LEW) and FISCHER (F344) rats, which exhibit opposite susceptibility to infections, autoimmune diseases and experimentally induced tumors. LEW rats develop strong Th1-pro-inflammatory responses in opposition to F344 rats which show a low to moderate Th1-pro-inflammatory profile. This opposite susceptibility and polarization has been linked to different basal levels of CORT (lower in LEW rats) [19,20,22]. In humans, baseline epinephrine (EPI) levels condition cytokine responsiveness and, through this mechanism, intrinsically hypo- or hyper-active adrenal medullas may shape opposite cytokine profiles in some individuals [21]. To the best of our knowledge, immune, nervous and endocrine systems have never been studied together in avian species in order to disclose whether their interaction may lead to categorization of a population into different groups. The objective of our study was to determine the existence of INPs in avian species. In particular, we aimed to evaluate whether INPs may be present in a population of adult *Coturnix coturnix*. The study may have evolutionary implications for our understanding of the evolution of phenotypic diversity in mammals and birds that would help to comprehend whether the LEW/F344 INPs are categories restricted to mammals or if they may represent shared strategies with birds.

## Materials and Methods

### Animals and Husbandry

Quail (*Coturnix coturnix*) is a domesticated species housed in captivity and recognized as a useful laboratory model for avian studies. Also, it is an important agricultural species in several countries [23,24] and data obtained with quail are extrapolated to chickens and other commercially important poultry species [25–28].

Husbandry was performed according to standard laboratory procedures (see details bellow) in order to minimize the effects of external factors such as housing conditions, food, water, temperature, photoperiod, aggressions and pecking behavior. I) Housing: 75 mixed-sex quail hatchlings were randomly housed in three white wooden boxes measuring 90 x 45 x 60 cm (length x width x height). At 28 days of age, the animals were sexed by plumage coloration and wing-banded for later individual identification and remained in the same box until 60 birds were reallocated in couples (one male and one female) in adult home cages (day 42 of age). The number of 60 birds was reached by randomly selecting 20 animals (10 male and 10 female) from each box. The housing condition in adult home cages prevented male-male interactions, thus minimizing dominance conflicts. II) Food and water: A quail starter diet (28% CP; 2,800 Kcal ME/kg) and water were provided *ad libitum*. Coincident with banding, birds were given a laying ration (21% protein, 2,750 kcal ME/kg) and water continued *ad libitum*. Each box had one feeder and 8 automatic nipple drinkers. A wire-mesh floor (1 cm grid) was raised 5 cm to allow the passage of excreta and a lid prevented the birds from escaping. III) Temperature and photoperiod: Brooding temperature was 37.5°C during the first week of life, with a weekly decline of 3.0°C until room temperature (24–27°C) was achieved. Quail were subjected to a daily cycle of 14 h light (300 to 320 lx):10 h dark during the whole study. Lights were turned on at 06:00 and turned off at 20:00. The conditions previously detailed are described as optimal for quail development [12,29–32]. IV) Health status: Animals’ health status was monthly verified by the veterinarian of the Institute. V) General welfare: animals were weekly checked for physical indicators trough visual examination of their plumage and foot health status [33–35]. There were no records of sick animals or poor welfare during this study. The conditions in which birds were raised were aimed to minimize potential stress load that could have affected birds’ physiology, with the scope and limitations that laboratory studies with domesticated species such as quail may have.

### Sampling procedure

Phytohemagglutinin-P (PHA-P) and antibody response against sheep red blood cells (SRBC) were induced on day 1. PHA-P response was determined 24 h later and after one week, 0.75 ml of blood was obtained from each bird by braquial-vein puncture. The blood was conserved on ice and immediately processed in order to determine: CORT levels, antibody response against SRBC (both analyzed in plasma), frequency of leukocyte subpopulation distribution (FLD) and interferon-γ (IFN-γ), IL-1β, IL-4 and IL-13 mRNA expression levels (in total blood). The total time for each sampling manipulation was always less than 80 s in order to ensure quantification of basal CORT levels [36].

### Determinations

Based on previous reports of INPs in *Rattus norvegicus* (LEW/F344 rats) [22] and *Homo sapiens sapiens* [21], the following group of INE variables was evaluated: plasma CORT as a representative of the HPA axis; lymphoproliferative response to PHA-P, antibody response against SRBC and FLD as immunity effectors; and four different INE interplay mediators that favor the milieu’s polarization: IFN-γ and IL-1β (pro-inflammatory) and IL-4 and IL-13 (anti-inflammatory). 60 mixed-sex randomly chosen adult Japanese quail were used to determine their response in the mentioned variables. To avoid possible variability induced by differences in sexual development and sex hormones, sampling procedures were conducted in same-age adult birds, that showed the following indicators of sexual maturity: stabilization of egg laying (females) and cloacal gland of at least 1000 mm^3^ plus positive foam production (male) [29,37,38].

- As a measure of cell-mediated immunity, the response to PHA-P injection, a lectin from *Phaseolus vulgaris* (Sigma Chemical, St Louis, MO, USA) was measured following methods described elsewhere [39–41]. Briefly, on day 1 a 0.05 ml intradermal injection of a 1 mg/ml solution of PHA-P in phosphate-buffered saline (PBS) was given in the wing web of each bird. The dermal swelling response was measured as the percentage of increase in wing web thickness at the injection site 24 h post-PHA-P. Measurements were recorded to the nearest 0.01 mm using a mechanical micrometer.
- Antibody response was assessed with a microagglutination assay [12,40,42]. Briefly, 20 μl of complement-inactivated plasma (through heating to 56°C) were serially diluted in 20 μl of PBS (1:2, 1:4, 1:8, up to 1:512). Next, 20 μl of a 2% suspension of SRBC in PBS were added to all wells. Microplates were incubated at 40°C for 1 h and hemagglutination of the test plasma samples was compared to the blanks (PBS only) and negative controls (wells with no SRBC suspension). Antibody titres were reported as log_2_ of the highest dilution yielding significant agglutination. The results represent the average of two duplicates for each animal.
- CORT concentrations present in plasma were quantified using a commercially available 125I corticosterone-radioimmunoassay (RIA) kit (MP Biomedicals, Costa Mesa, California, USA) developed for mice and used in similar studies with other animal species [43,44]. RIA was used following the procedure described in [43]. According to the manufacturers, cross-reactivity with other steroids was: desoxycorticosterone (0.34%), testosterone (0.10%), cortisol (0.05%), aldosterone (0.03%), progesterone (0.02%), and less than 0.01% for all other steroids tested. CORT concentration was expressed as nanograms of hormone per ml of plasma (ng/ml). To ascertain the biochemical validity of this assay, the following tests were performed: parallelism, accuracy and precision [43,45].
- To perform flow cytometry, 40 μl of total blood were stained with a fluorescent lipophilic dye (3, 3`-dyhexiloxacarbocyanine iodide; DiOC6, Molecular Probes) in order to obtain absolute counts of erythrocytes, lymphocytes, monocytes, thrombocytes and granulocytes as described elsewhere [46] (S1 Fig.) FLD number was calculated using the following formula: FLD = number of granulocytes/(number of lymphocytes + number of monocytes).
- To determine cytokine mRNA expression levels, 600 μl of total blood were processed as follows: chicken peripheral lymphocytes were isolated by gradient centrifugation in Histopaque 1077 (Sigma Aldrich Inc.) according to the manufacturer’s instructions and as previously described [47]. Total RNA was extracted from cells using TRIzol (Invitrogen, Carlsbad, CA) according to the manufacturer’s instructions also. The RNA was resuspended in 40 μl RNase-free water and quantified using a NanoDrop spectrophotometer (Biotek Sinergy HT). Then, approximately 1 μg of total RNA was reverse-transcribed in a final reaction volume of 20 μl containing the following components: 7X gDNA Wipeout buffer, 5X Quantiscript RT Buffer, Quantiscript Reverse Transcriptase, and RNase-free water. The resulting cDNA was stored at -80°C until use for real-time PCR. For quantitative real-time PCR assays, specific primers for quail IL-1β, IL-4, IL-13 and IFN-γ genes were used as previously reported [48] (S1 Table). β-actin was used as a reference housekeeping gene. Real-time PCR was performed with a Step One Plus Detection System (Real-Time PCR System-Thermo Fisher Scientific). The real-time PCR reaction mixture contained 1.0 μl of sample cDNA, 1.0 μl of forward and reverse primers (10 μM each), 5 μl of iQ SYBR Green Supermix (Bio-Rad) and 3.0 μl of nuclease-free water. A typical thermal profile consisted of one cycle of 10 min of polymerase activation at 95°C, followed by 40 cycles of PCR at 95°C for 15 s and specific annealing temperature for 60 s [48]. Expression of the target genes was measured relative to that of β-actin. The results represent the average from three technical replicates for each analyzed animal. The level of expression of each target gene was calculated using the formula: Gene Level = 2^-(Target Gene Ct – β-Actin Ct)^ The value obtained was used to compare the level of expression of each molecule [49].

### Statistical Analysis

Multivariate statistic (Principal Component Analysis (PCA)) was performed to explore and describe general data variability, using the following explanatory variables: plasma CORT, lymphoproliferative response to PHA-P, antibody response against SRBC and FLD. Correlation analysis was then performed in order to complement the information provided by the PCA. One way-ANOVAs were used to compare differences on basal CORT levels, lymphoproliferative response to PHA-P, antibody response against SRBC and FLD. Differences in pro and anti-inflammatory mediator profiles (IFN-γ, IL-1β, IL-4 and IL-13) were evaluated using a generalized linear model analysis assuming a Gamma distribution and a Correlation analysis was also used to explore relationship between CORT and the molecular mediators. In every case, a *p*-value < 0.05 was considered to represent significant differences. All statistical analyses were performed using InfoStat [50].

### Ethic Statement

The study complies with all applicable Argentinian laws, with the local Argentinian Association for Science and Technology Laboratory Animals – (AACyTAL Bulletins number 15 and 16, 2001) and it was approved by the Institutional Committee for Care and Use of Laboratory Animals of the Facultad de Ciencias Exactas, Físicas y Naturales, Universidad Nacional de Córdoba, Argentina. All experiments were carried out in accordance with the National Institute of Health Guide for the care and use of laboratory animals (NIH Publications No. 80–23, revised 1996).

## Results and Discussion

**Fig 1:**
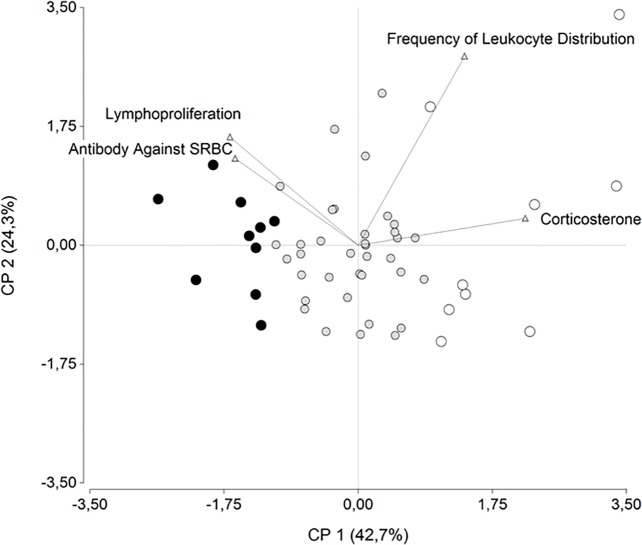
Exploration of data variability. Principal Component Analysis Bi-plot graph. Each dot represents an animal in the study and triangles represent the explanatory variables used in the analysis. Full white dots (○) and full black dots (●) represent extreme low and high CORT birds respectively. The eigenvalues of each PC are shown in brackets next to each component.

Multivariate analysis shows that the Principal Component 1 (PC1) explains the 43% of total data variability (eigenvalue = 0.43). The remaining eigenvalues for the other principal components were of 0.24 (PC2), 0.20 (PC3) and 0.13 (PC4), completing the 100% of the data variability. Analyzing the influence of each variable in the configuration of PC1, CORT has an eigenvector of 0.63 (Fig. 1). This hormone is the variable with the highest eigenvector in the analysis; this implies that CORT presents the highest positive influence on PC1 configuration.

In order to properly characterize the relationship between variables a Pearson correlation was used Table 1. The analysis informed that the only variable that significantly correlated with the others is CORT, showing significant positive correlations with FLD (0.34) and negative correlations with Lymphoproliferation and Ab response against SRBC (-0.35 and -0.31).

**Table 1:** Pearson correlation analysis between CORT and immunity effectors.

	Corticosterone	Lymphoproliferation	Antibody Against SRBC
**Lymphoproliferation**	−0,35**		
**Antibody Against SRBC**	−0,31*	0,21	
**FLD**	0,34*	−0,06	−0,09

* and **, r value significant at p < 0.05 and 0.01 respectively. The r values for every variable where obtained from 60 birds.

**Fig 2:**
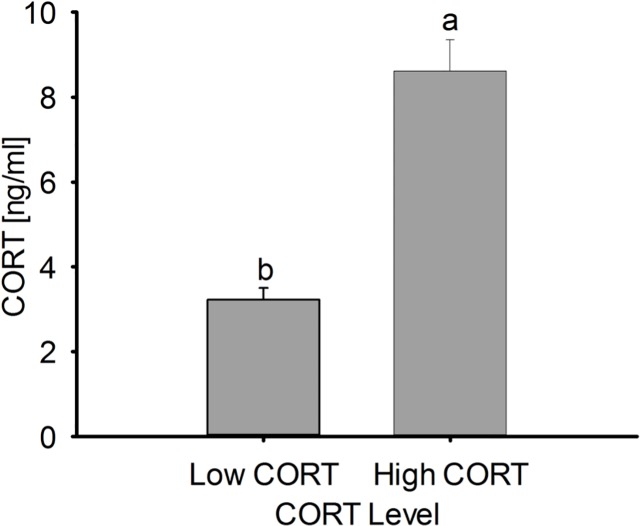
Determination of divergent basal CORT level groups. Low and High CORT animals grouped based on their CORT level. Birds belonging to the High CORT group have 2.7-fold higher CORT concentrations than their Low CORT counterparts. Data are means ± SE. Different letters indicate significant (*p* < 0.05) differences between groups. Number of birds in the study = 60, number of birds per group = 10.

Together, our results show that CORT is the most influential variable in the explanation of the variability between animals and that correlates with all the other variables measured. It is also important to highlight that this hormone (i) is a marker of HPA neuroendocrine axis activity which is essential in immune-neuroendocrine interactions [16,51–53], (ii) acts as a powerful endogenous immune modulator [12,13,17,47,53,54] and (iii) is the central hormone involved in the LEW/F344 paradigm [19,22], which turned out to be the first INP outcome in mammals. Therefore we used CORT to delimitate two extreme groups of animals that could express putatively divergent phenotypes. According to their CORT basal level and their PCA bi-plot graph distribution, birds within the top and bottom 16% extremes of the population were designated as High and Low CORT, respectively (see further details below). Elenkov et al. (2008) determined INPs in other species by subtracting and adding 1 standard deviation from the mean of the grouping hormone population value. In our study, all the birds assigned to the high or low CORT group were also found to show individual values differing at least 1 standard deviation from the population mean. ANOVA consequently revealed a highly significant main effect of CORT level (*F*_1,18_ = 40, *p* < 0.001) (Fig. 2). The two groups previously defined were hence denominated “Low CORT” or “High CORT” (animals with the lowest or the highest hormonal levels, respectively). Animals belonging to the High CORT selected group had on average 2.7-fold higher CORT concentration than their Low CORT counterparts.

High and Low CORT groups were estimated based on the basal animal hormone level. However, handling of the birds, sampling order or even their gender could have affected CORT determination [4,12,25]. Total time for each sampling manipulation was always less than 80 s minimizing animal manipulation and avoiding handling effects on CORT determination [36]. A correlation analysis between sampling order and CORT values showed no significant influence, and no gender effects were detected. Other factors that could affect CORT concentration such as housing, food, water, temperature, photoperiod, health status, welfare, aggressions and pecking behavior (see material and methods) were also controlled. Altogether these suggest that CORT levels, as well as other variable responses determined, were not reflecting potential effects derived from the bird´s manipulation or stressors.

**Fig 3:**
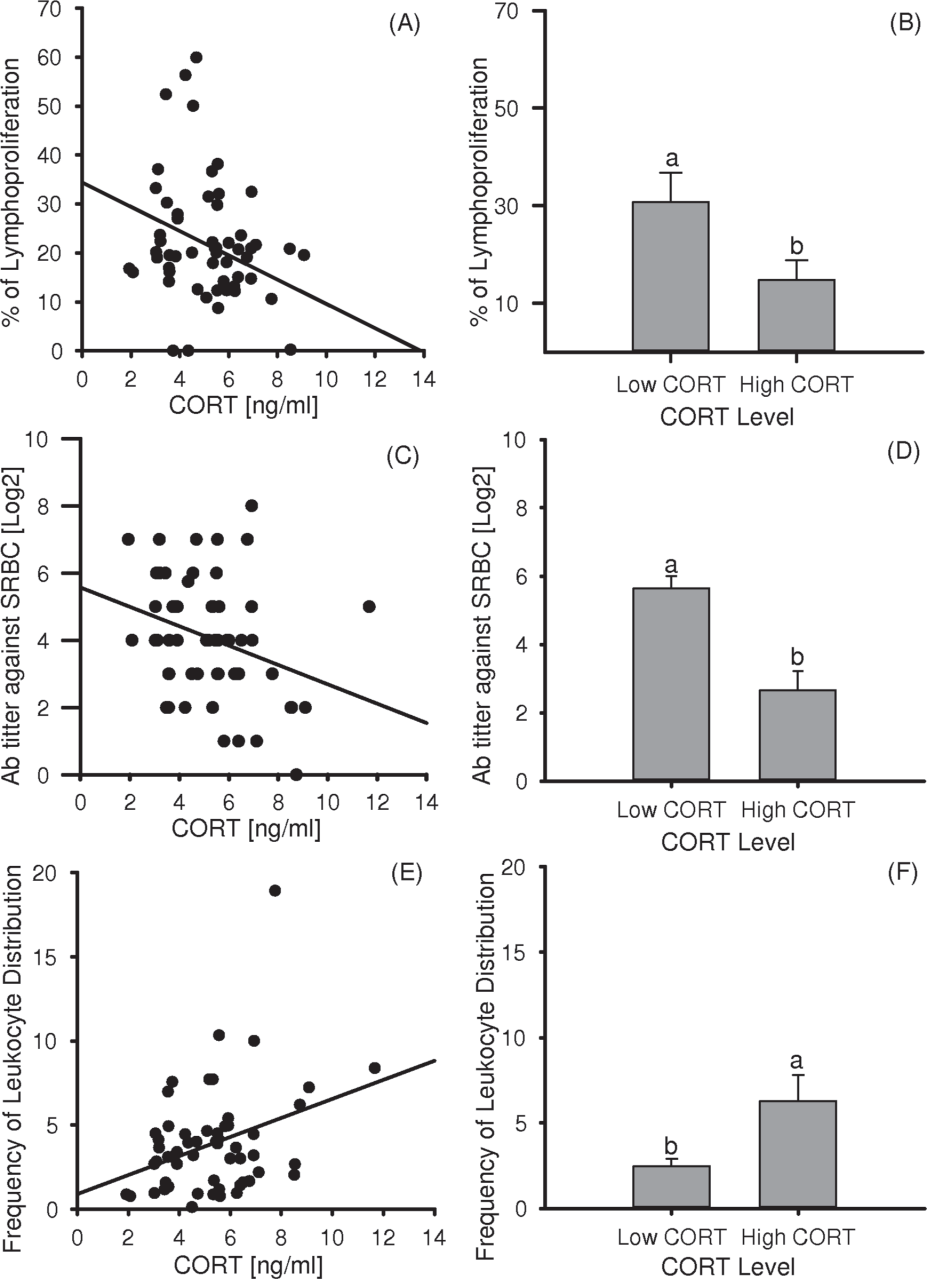
Immune effector analysis in divergent Low and High CORT groups. Correlation plots between CORT and lymphoproliferative response to PHA-P, antibody response against SRBC and FLD are presented in panels A, C and E. Effect of the aggrupation dependent on the basal levels of CORT on immune effectors is shown in panels B (lymphoproliferative response to PHA-P), D (antibody response against SRBC) and F (FLD). Data are means (number inside bars) ± SE. Number of birds per group = 10. Different letters indicate significant (*p* < 0.05) differences between groups. FLD number was calculated using the following formula: FLD = number of granulocytes/(number of lymphocytes + number of monocytes).

Immune effector analysis confirms and complements the information obtained via the correlation analysis (see Fig. 3 for correlation plots and extreme groups bar graphs): quail with lowest CORT levels showed higher lymphoproliferative swelling responses to PHA-P (*F*_1,18_ = 4.97, *p* = 0.03; Fig. 3 A and B), higher antibody response against SRBC (*F*_1,18_ = 18.9, *p* < 0.001; Fig. 3 C and D), and lower FLD (*F*_1.18_ = 5.94, *p* = 0.024; Fig. 3 E and F) than their high CORT counterparts. Thus, a higher PHA response, a marker of T lymphocyte proliferation and an indicator of constitutive and non-specific immunity (phagocytosis by heterophils and monocytes) [55], was linked to a higher humoral immune response to the T-dependent antigen SRBC. According to previous reports [56], adrenocorticotropic hormone and CORT are immunosuppressive and adrenocorticotropic hormone/glucocorticoids axis induction generally reduces lymphocyte and increases heterophil numbers [57–59]. This phenomenon could explain the different leukocyte subpopulation distribution found in the two extreme CORT groups, and at the same time explains the correlation found in the analysis. Findings imply that animals differing in their basal CORT levels also manifest dissimilar and opposite immune effector responses.

**Fig 4:**
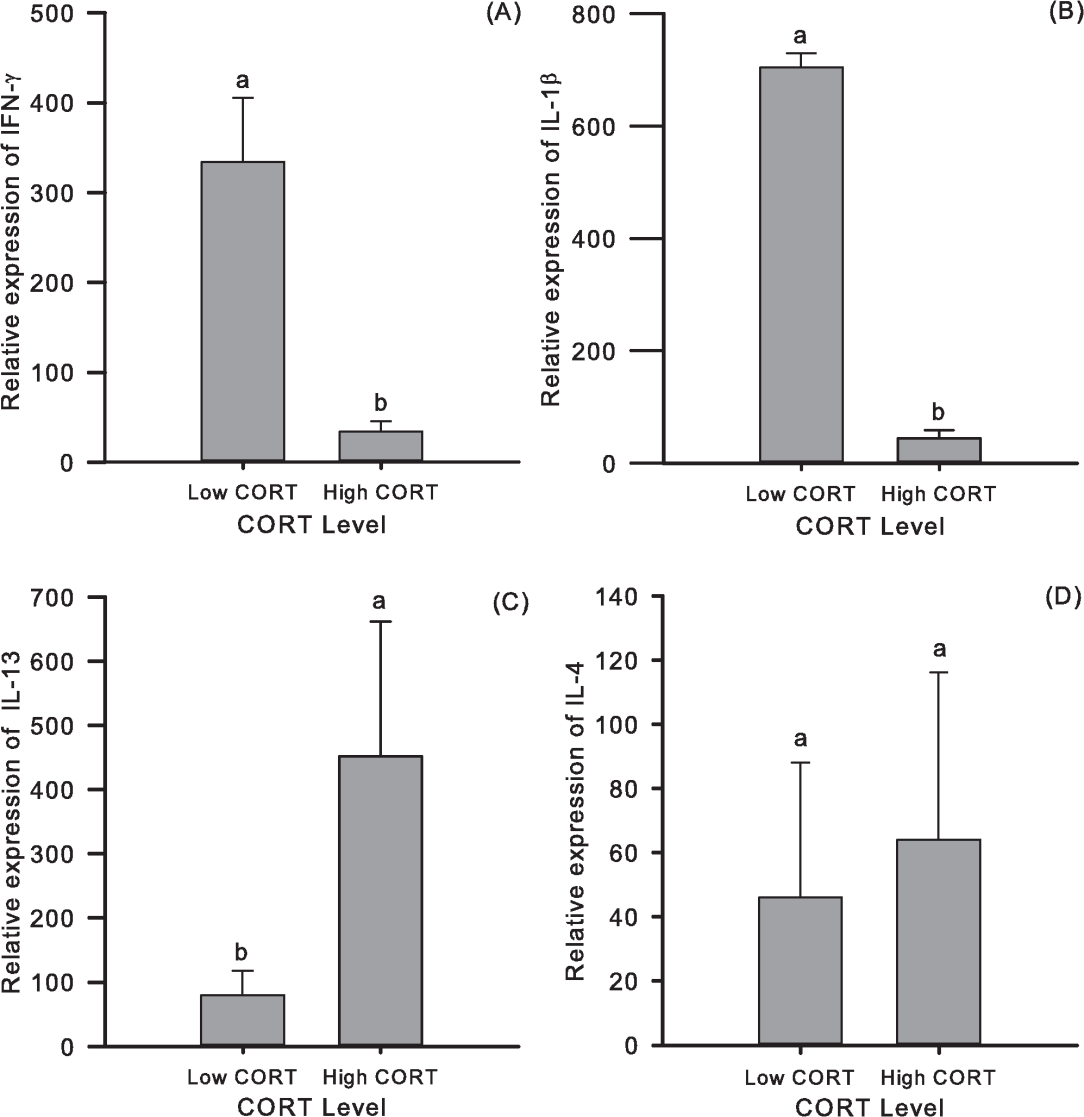
Analysis of molecular mediators in divergent Low and High CORT groups. Effect of the aggrupation dependent on the basal levels of CORT on the expression of two pro-inflammatory (IFN-γ and IL-1β, panels A and B) and two anti-inflammatory mediators (IL-13 and 4, panels C and D). Data are adjusted means (number inside bars) ± SE. Number of birds per group = 10. Different letters indicate significant (*p* < 0.05) differences between groups. The relative expression of each target gene was calculated using the formula: Gene Level = 2^-(Target Gene Ct – β-Actin Ct)^. The value obtained was then multiplied by 1x10^8^ in order to fit the scale of the graphs.

The mRNA expression levels of two pro-inflammatory (IFN-γ and IL-1β) and two anti-inflammatory cytokines (IL-4 and IL-13) between Low and High CORT birds were studied in order to reveal if they also exhibited a molecular substrate underlying the putative phenotypes. Expression levels were evaluated with a generalized linear model analysis assuming a Gamma distribution. The analysis of these molecular mediators revealed that opposite groups differed in three of the four mediators tested (Fig. 4). Animals exhibiting low basal CORT showed significantly higher mRNA expression levels of IFN-γ (9.8 fold; Fig. 4 A) and IL-1β (15.6 fold; Fig. 4 B) and lower levels of IL-13 (5.7 fold; Fig. 4 C) in comparison with their high CORT counterparts.

A Spearman correlation analysis was then performed in order to inform about the relationship between CORT and the new variables introduced. The correlation analysis done informed that 3 of the 4 variables significantly correlate with CORT Table 2. IFN-γ and IL-1β are negatively correlated (-0.75 and -0.72 respectively) and IL-13 shows a positive correlation (0.72) with the hormone. Significant correlations between IFN-γ and IL-1β (0.44), as well as negative correlation between IL-1β and IL-13 (-0.45) confirm previously published information concerning mediator interaction in avian species [56].

**Table 2:** Spearman correlation analysis between CORT and molecular immuno-neuroendocrine mediators.

	Corticosterone	IFN-γ	IL-1β	IL-13
**IFN-γ**	−0,75*			
**IL-1β**	−0,72*	0,44*		
**IL-13**	0,72*	−0,39	−0,45*	
**IL-4**	−0,28	0,08	−0,45	−0,45

*, r value significant at p < 0.01. The r values for every variable where obtained from 20 birds.

It is worth mentioning that those animals with low CORT levels had a polarized cytokine milieu, where pro-inflammatory mediators predominated over anti-inflammatory ones. Up to this point, this milieu (concerning IFN-γ, IL-1β and IL-13 levels) as well as the response of effector variables (lymphoproliferation, antibody response against SRBC and FLD) seems to be dependent on the animals’ CORT basal level. Different consequences may follow from this fact, mainly related to risk or resistance to certain immune-neuroendocrine challenges in a wide spectrum, from stress response *per se* to immune-related diseases.

In order to characterize the immuno-neuroendocrine response from the resulting different subsets of animals the number of birds in each extreme was gradually increased from 10 to 20% of the total population. The sub-set equal to the 16% of the animals mentioned above allowed a clear assignation of each of the birds to either one or other extreme phenotypes proposed. It is important to highlight that if that percentage is increased above the 16%, although CORT remains different in all animals that fit within the new two extreme setups, not all the remaining variables allow a clear fitting of the new subset of birds within the proposed Lewis-Fischer like profiles. In particular we start losing discrimination power on the lymphoproliferative response variable. Our findings are in line with previous cutoff proposed for the description of the INPs phenomenon in humans and rats (about 15% in both cases) [19,21].

**Fig 5:**
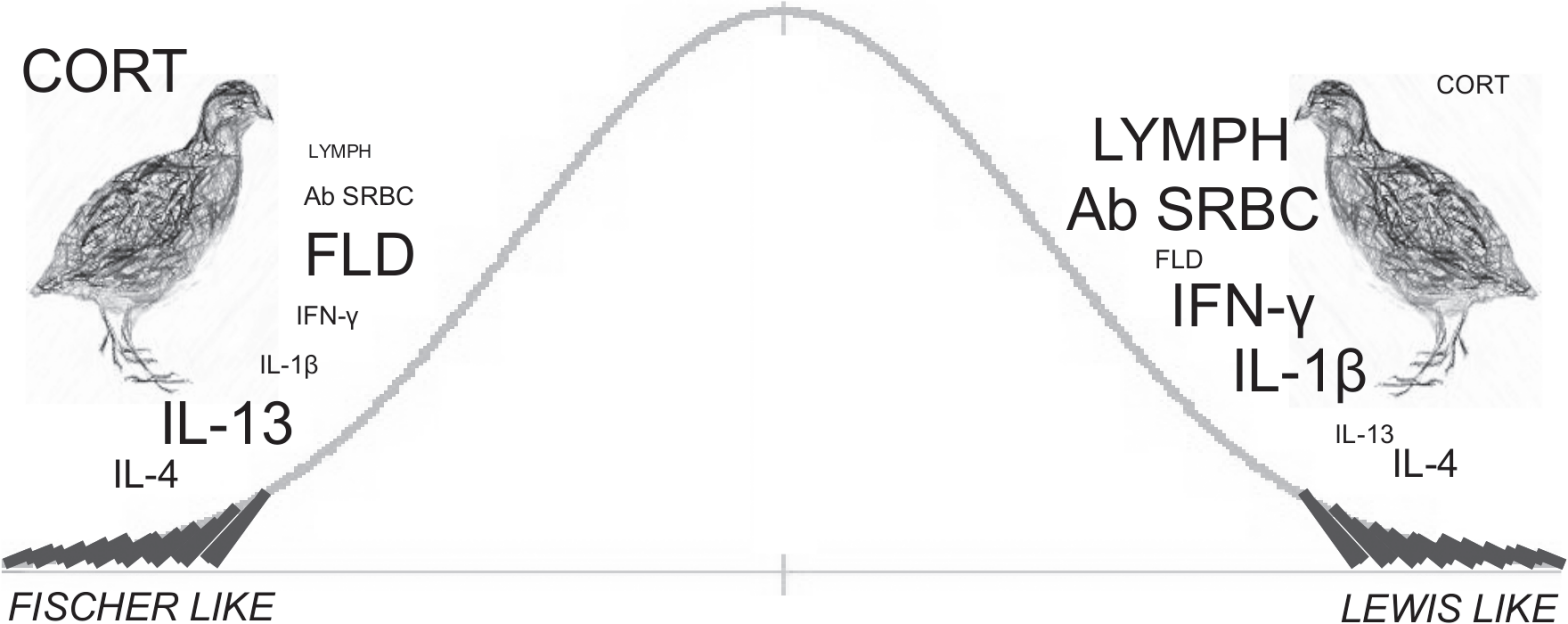
Schematic conceptual representation of INPs in *Coturnix coturnix*. The variables set to determine the existence of avian INPs in the present study are represented around each bird. The size of the variable indicates if the animals show high or low response in each of the parameters in the INPs. LYMPH: lymphoproliferative response to PHA-P; Ab SRBC: antibody response against SRBC; FLD: frequency of leukocyte distribution; level of expression of mediators: IFN-γ and IL-1β (pro-inflammatory); and IL-4 and 13 (anti-inflammatory). "Fischer-like" quail with high CORT levels also manifest high FLD and IL-13, but low LYMPH, Ab SRBC, IFN-γ and IL-1β levels. "Lewis-like" counterparts have low CORT as well as low FLD and IL-13 responses, but high LYMPH, Ab SRBC, IFN-γ and IL-1β responses. These two extreme groups of birds do not differ in their IL-4 level.

Elenkov et al. [21] identified two subgroups in healthy human individuals with relatively low and high EPI outputs that had opposite innate cytokine profiles, providing a clear link with the LEW/F344 paradigm. Our study results are similar in the following aspects: two groups of quail had high or low basal CORT levels and opposite innate cytokine profiles, and ergo divergent effector responses (Fig. 5). These findings may extend the scope to which the INP notion could be applied and considered in future studies. In this sense, our work may represent a starting point and several questions still remain to be answered. For example, are these two opposite INPs distributed in more avian species? Has the expression of the INPs a relation with the level of domestication of the specie? Apart from CORT, are other mediators involved in the configuration of INPs in birds? How stable or consistent are INPs along the ontogeny of an animal? Could the CORT difference be a result of a genetic difference in hypothalamic secretion of corticotrophin releasing hormone as happened in the LEW/F344 paradigm? Since LEW and F344 rats have opposite susceptibility to experimental immunological diseases, are the analogous phenotypes in birds linked to differing responsiveness or susceptibility to infections and immune-related diseases?

The implications of INPs as a phenomenon in an avian species could be very important as an evolutionary strategy. The INPs described suggests the existence of equally fit animals in an adult bird group with different arrangements in their INE interactions. The advantage of different and coexistent INPs may be an increased ability to deal as a group with a wide range of challenges demanding plastic responses in the INE interplay context. In this sense, varied phenotypes within a group may imply the possibility of showing different responses to a challenge, whereas a homogeneous population expressing only one INP may find this possibility limited. At the same time, it is also plausible that contemporary quail populations may be the result of their ancestors’ response to domestication. In this sense, there is a possibility of considering the INPs described as representatives of a physiological compromise between the specie needs and the pressures imposed by domestication processes.

A significant increase in the study of cytokines, hormones and neurotransmitters, as well enormous progress in understanding the biology of immune, nervous and endocrine systems, has provided major advances in the understanding of INE interactions [1–9,15,59,60]. Our study described, in a population of 60 Japanese quail, two groups of birds comparable to their mammalian counterparts in the LEW/F344 INE interaction paradigm (Fig. 5) [21,22]. The “LEW-like quail” showed low CORT levels together with high levels of pro-inflammatory mediators and a Th1-like response, whereas the “FISCHER-like quail” showed the opposite configuration. This suggests for the first time that these INPs are not restricted to the mammalian species studied to date (*Homo sapiens sapiens* and *Rattus norvegicus*) [19,21,22]. Higher vertebrates had a common reptilian ancestor more than 200 million years ago. We cannot make evolutionary judgments based on the results obtained herein because the methods limit us and it would be too pretentious. However, we consider it worth proposing that INPs could have been shared as a physiological strategy in three species belonging to two different evolutionary lineages. Future studies may confirm whether INPs are a shared strategy between mammals, birds and their reptilian ancestor or if they are independent outcomes of the interactions between the immune, nervous and endocrine systems in each lineage.

## Supporting Information

S1 Fig Flow cytometry dot plot. (DOC)

S1 Table Primer sequences for real-time PCR amplification of quail target genes. (DOC)
