# Supplementary material for: Immune Neuroendocrine Phenotypes in Coturnix coturnix: Do Avian Species Show LEWIS/FISCHER-Like Profiles?
Source: PLoS One. 2015 Mar 20;10(3):e0120712. doi: 10.1371/journal.pone.0120712 (PMC4368694; doi:10.1371/journal.pone.0120712)
Supplement: S1 Fig — (DOC) [file pone.0120712.s001.doc]

**S1_Figure**: Flow cytometry dot plot.

Dot plot showing the different leukocytes subpopulations involved in the determination of the Frequency Leukocytes Subpopulation Distribution (FLD).

## 
