# Supplementary material for: Immune Neuroendocrine Phenotypes in Coturnix coturnix: Do Avian Species Show LEWIS/FISCHER-Like Profiles?
Source: PLoS One. 2015 Mar 20;10(3):e0120712. doi: 10.1371/journal.pone.0120712 (PMC4368694; doi:10.1371/journal.pone.0120712)
Supplement: S1 Table — (DOC) [file pone.0120712.s002.doc]

**S2_ Table**: Primer sequences for real-time PCR amplification of quail target genes. IFN: interferon, IL: interleukin.

| | Target gene | Primer sequence (5′–3′) | Accession number | | --- | --- | --- | | IFN-γ | F: CAACCTTAATGATGGCACGA  R: CTTTGCGGTGGATTCTCA | AJ001678 | | IL-1β | F: CTTCCTCCAGCCAGAAAGT  R: CAGCTTGTAGCCCTTGAT | AB559570 | | IL-4 | F: GAGAGCATCCGGATAGTGAAG  R: TTCGCATAAGAGCTGGGTTC | AB559571 | | IL-13 | F: CTGCAAGAAGGACTATGAGCCC  R: CAGTGCCGGCAAGAAGTT | AB5595764 | | β-Actin | F: CTGGCACCTAGCACAATGAA  R: CTGCTTGCTGATCCACATCT | AF199488 | |  | | | | | | | |
| --- | --- | --- | --- | --- | --- | --- | --- | --- | --- | --- | --- | --- | --- | --- | --- | --- | --- | --- | --- | --- | --- | --- | --- | --- | --- | --- |
|  |  |  |  |  |  |  |  |  |
